# A factor analysis model for functional genomics

**DOI:** 10.1186/1471-2105-7-216

**Published:** 2006-04-21

**Authors:** Rafal Kustra, Romy Shioda, Mu Zhu

**Affiliations:** 1 Public Health Sciences, Health Sciences Bldg, University of Toronto, Toronto, ON, Canada; 2 Department of Combinatorics and Optimization, University of Waterloo, Waterloo, ON, Canada; 3 Department of Statistics and Actuarial Science, Universityof Waterloo, Waterloo, ON, Canada

## Abstract

**Background:**

Expression array data are used to predict biological functions of uncharacterized genes by comparing their expression profiles to those of characterized genes. While biologically plausible, this is both statistically and computationally challenging. Typical approaches are computationally expensive and ignore correlations among expression profiles and functional categories.

**Results:**

We propose a factor analysis model (FAM) for functional genomics and give a two-step algorithm, using genome-wide expression data for yeast and a subset of Gene-Ontology Biological Process functional annotations. We show that the predictive performance of our method is comparable to the current best approach while our total computation time was faster by a factor of 4000. We discuss the unique challenges in performance evaluation of algorithms used for genome-wide functions genomics. Finally, we discuss extensions to our method that can incorporate the inherent correlation structure of the functional categories to further improve predictive performance.

**Conclusion:**

Our factor analysis model is a computationally efficient technique for functional genomics and provides a clear and unified statistical framework with potential for incorporating important gene ontology information to improve predictions.

## Background

Functional genomics is often described as one of the most important challenges in the post-genomics era. Now that many genomes have been sequenced, the next step is to understand the functions of all the gene-products. One of the most accessible genome-wide modalities that has proven useful for this task is expression microarray experiment e.g., [1-3]. The general idea is to associate genes to functional categories by comparing their expression profiles to genes with known functional associations. This is usually supported by the biological argument that genes in the same or similar functional categories are often part of a functional pathway and therefore likely to be co-regulated by the same set of transcription factors.

In a typical functional genomic experiment relying on expression data, a few dozen to a few hundred samples from an organism are processed and hybridized. The samples may be obtained from different tissues and organs, e.g., the mouse experiment of [3], or from different experimental conditions [1]. The expression data for *p *genes are then assembled in a *p *× *n *matrix where each row presents an expression profile of the corresponding gene across *n *samples.

A few distinct ways have been proposed to predict gene functions from microarray data. The authors of [1] run a multitude of clustering methods which generate thousands of (overlapping) clusters. For each functional category, every cluster is assigned an *enrichment *p-value based ona hypergeometric distribution. Those clusters with p-values below a certain threshold are used for prediction: an uncharacterized gene is localized among all enriched clusters and predicted to be involved in all functions these clusters entail. This is biologically sensible since a gene is usually involved in multiple functions. The authors of [2] use gene-gene correlations to build graphical models of gene expression data which are then used, among other things, for functional prediction. The predictions are done by first forming a set of shortest paths between genes, thresholded at some length to retain significance, and predicting with a constrained majority rule using the genes on the path.

One model-based approach, perhaps more intuitive for statisticians, is to use one of many classification methods. In such an approach, one starts with the expression matrix, where genes correspond to observations and their expression profiles correspond to features. A set of *K *functional categories is specified which span all interesting and relevant biological functions for a given genome. Since any given gene can belong to multiple functional categories, the simplest approach is to build *K *separate binary classifiers, one for each function, and use them to predict whether each of the uncharacterized genes belongs to one of the *K *functional categories.

This general paradigm permits one to use many known classification models and algorithms. The authors of [3] apply the Support Vector Machine (SVM) classifier, popular in the machine learning literature, to a set of 55 microarrays of different mouse tissues to predict functions of over 12,000 mouse genes which currently have unknown status. They use a set of 992 functional categories defined by Gene Ontology – Biological Process (GO-BP; see [4]), which is a hierarchical categorization of functions much finer in scale than the set of 42 YPD Cellular Role functions, largely independent of each other (in the sense that most genes belong to at most two functional categories), which was used by [1]. More details about GO-BP are given below.

It is important to acknowledge that functions of gene-products cannot, in many cases, be fully resolved from co-expression data alone: co-expression may be a result of other factors besides co-regulation, e.g., separate pathways that happened to be activated in the same experiment, or simply chance. Similarly, trans-regulation factors may be acting more globally inducing co-expression of genes that are weakly connected or completely disconnected on a functional level. Some of these concerns can be alleviated by using expression data that represent a wide array of conditions, so that significant observed co-expression between genes is more likely to have resulted from close functional relationship of respective gene-products than from other factors. Studies with such design, as those already cited [1,3], have been successful in that regard. As with many other genome-wide studies that use high-throughput technologies, this proposal can potentially be highly useful as an initial hypothesis-generating step.

In this paper, we propose a new approach to predicting gene functions using expression data and GO-BP annotations. It is worth noting that our framework can, in principle, be extended to utilize further sources of information, besides expression data (see "Discussion" section). Such approaches, sometimes dubbed "genomic data fusion" models, have been proposed in the literature and show some promise of improving prediction accuracy. In [5], the authors utilize phylogenetic profiles and fused domain methods in addition to experimental data that include mRNA expression to derive a large number of candidate protein-protein links and hence predict function in yeast *Saccharomyces cerevisiae*. A method which is similar in spirit but more refined in terms of an approach to assigning interactions from various genomic sources, has been proposed by [6], who use phenotype data from gene knockout experiments, cellular localization data, and information from protein-protein interaction databases in addition to expression data. Each data source contributes a bit matrix that codes all the protein-protein relationships. Depending on the manner of inferring the relationships (e.g., various p-value thresholds of Pearson correlation coefficient for expression data), and on the manner in which various bit matrices are combined, one can control the contribution of each data source and arrive at the final association matrix and hence predict gene functions. A moremodel-oriented approach to data fusion is described in [7] and [8], which propose the Bolztmann machine and SVMs, respectively, for gene-product function prediction using various genomic data sources.

### Gene Ontology – biological processes

We use the GO-BP annotations to provide us with a set of functional categories and the associations of characterized genes to these categories. Gene Ontology [9] is a popular information resource for genetic research. The Biological Process part (GO-BP), which is relevant for the expression data, has currently almost 10,000 processes arranged in a Directed Acyclic Graph (DAG) structure. The top node in this DAG (Biological Process, accession number GO:0008150) has six children (Development, Behaviour, Physiological Process, Viral Life Cycle, Cellular Process, Regulation of Biological Process) which constitute major groupings of biological processes in all organisms. Each of these is subdivided into more specialized characterizations. The seventh top child is a Biological-Process Unknown node, which has no further descendants; it is applied to currently uncharacterized genes. The arcs in the GO-BP DAG usually denote subdivisions of a process characterization (commonly called "is_a" relationships), although sometimes they also denote partitions of a process into smaller steps (commonly called "part_of" relationships). The GO-BP is a cross-organism ontology; for any specific organism only a part of its structure will usually be relevant. For example, for simple eukaryotes such as yeast studied in [1], the whole subgraphs under Viral Life Cycle and Behaviour are not relevant and thus have no genes associated to them.

In addition to providing a continually evolving ontology structure, the GO consortium also maintains and curates the association data. For most model organisms, one can obtain a table where each known gene or Open Reading Frame (ORF) is associated with a set of biological processes. These associations are built and maintained based on reliable evidence such as major publications or directly confirmable laboratory experiments, although there is a range of "credibility" scores. These scores are called Evidence Codes and are maintained alongside the association data. They could potentially be used in the analysis to weigh different associations according to their credibility, but we have chosen not to make this distinction in this research.

### Motivation and summary of our study

A problem with the typical classification approach is its need to train very large numbers of classifiers on thousands of genes. This is further complicated by the need to use multiple cross-validation experiments for each classifier to fine tune its control parameters. These requirements are computationally prohibitive for most laboratories. The authors of [3], for example, had access to a powerful computer cluster to perform extensive computations, an atypical resource in many biological labs.

We propose a factor analysis model (FAM) to predict functions for uncharacterized genes using microarray expression data and a subset of the GO-BP functional categories. We show that our factor analysis approach provides significant computational advantages without compromising prediction accuracy. It is worthwhile to notice that FAM directly models the expression-induced correlation structure among genes. This correlation structure coupled with small signal-to-noise ratios of microarray data, can negatively impact the statistical efficiency of some classifiers that treat genes as independent observations [10,11]. Finally, we show that our model has the potential for incorporating important gene ontology information and deal with a number of major challenges in using statistical classification techniques for functional genomics (see "Discussion" section).

## Results

This section gives an overview of our prediction model, experiments (including descriptions of our data, preprocessing steps, benchmarks and performance evaluation metrics) and computational results.

### The model

Our model, FAM, applies factor analysis to predict gene functions using microarray data.

#### The typical approach

We first describe the typical computational approach, e.g., [3], more formally. Let **Z **be the *p *× *n *matrix of gene expression data; the (*i*,*j*)^th ^element of that matrix, *z*_*ij*_, is the expression level of gene *i *from experiment (or sample) *j*. Without loss of generality, we shall assume that the GO-BP data contain the functional annotations of the first *p*_1 _<*p *genes. In particular, with a total of *K *different functional categories, this information can be stored in a binary *p*_1 _× *K *matrix, which we call **Y**_1_. Its (*i*, *k*)^th ^element *y*_*ik *_equals 1 if gene *i *is associated with function *k*, and equals 0 otherwise. We use **Y**_2 _to denote the unknown functional annotation matrix for the remaining *p*_2 _= *p *- *p*_1 _genes; thus **Y**_2 _is the *p*_2 _× *K *matrix that needs to be predicted.

Using the matrix **Y**_1 _and the first *p*_1 _rows of **Z**, we can build predictive models to associate gene functions (**Y**) from the microarray data (**Z**). Typically, *K *different models {*F*_*k *_: *k *= 1, 2, ..., *K*} are constructed independently, one for each functional category, i.e., each column of **Y**. Since the elements of **Y**_1 _are binary, each model *F*_*k *_is simply a classifier. In theory, any classification algorithm can be used here, the most popular being SVMs, e.g., [3]. Each model *F*_*k *_is then applied to the remaining *p*_2 _rows of **Z **to predict whether or not these genes are associated with function *k*.

#### The factor analysis model (FAM)

Let g1(j),g2(j),…,gp(j)
 MathType@MTEF@5@5@+=feaafiart1ev1aaatCvAUfKttLearuWrP9MDH5MBPbIqV92AaeXatLxBI9gBaebbnrfifHhDYfgasaacH8akY=wiFfYdH8Gipec8Eeeu0xXdbba9frFj0=OqFfea0dXdd9vqai=hGuQ8kuc9pgc9s8qqaq=dirpe0xb9q8qiLsFr0=vr0=vr0dc8meaabaqaciaacaGaaeqabaqabeGadaaakeaacqWGNbWzdaqhaaWcbaGaeGymaedabaWaaeWaaeaacqWGQbGAaiaawIcacaGLPaaaaaGccqGGSaalcqWGNbWzdaqhaaWcbaGaeGOmaidabaWaaeWaaeaacqWGQbGAaiaawIcacaGLPaaaaaGccqGGSaalcqWIMaYscqGGSaalcqWGNbWzdaqhaaWcbaGaemiCaahabaWaaeWaaeaacqWGQbGAaiaawIcacaGLPaaaaaaaaa@410B@ be the expression level of *p *different genes from the *j*^th ^microarray experiment. Using a factor analysis model [[12], Chapter 9], we model the expression level gi(j)
 MathType@MTEF@5@5@+=feaafiart1ev1aaatCvAUfKttLearuWrP9MDH5MBPbIqV92AaeXatLxBI9gBaebbnrfifHhDYfgasaacH8akY=wiFfYdH8Gipec8Eeeu0xXdbba9frFj0=OqFfea0dXdd9vqai=hGuQ8kuc9pgc9s8qqaq=dirpe0xb9q8qiLsFr0=vr0=vr0dc8meaabaqaciaacaGaaeqabaqabeGadaaakeaacqWGNbWzdaqhaaWcbaGaemyAaKgabaWaaeWaaeaacqWGQbGAaiaawIcacaGLPaaaaaaaaa@3271@ as

gi(j)=∑l=1Lλilfl(j)+εi(j),     (1)
 MathType@MTEF@5@5@+=feaafiart1ev1aaatCvAUfKttLearuWrP9MDH5MBPbIqV92AaeXatLxBI9gBaebbnrfifHhDYfgasaacH8akY=wiFfYdH8Gipec8Eeeu0xXdbba9frFj0=OqFfea0dXdd9vqai=hGuQ8kuc9pgc9s8qqaq=dirpe0xb9q8qiLsFr0=vr0=vr0dc8meaabaqaciaacaGaaeqabaqabeGadaaakeaacqWGNbWzdaqhaaWcbaGaemyAaKgabaWaaeWaaeaacqWGQbGAaiaawIcacaGLPaaaaaGccqGH9aqpdaaeWbqaaGGaciab=T7aSnaaBaaaleaacqWGPbqAcqWGSbaBaeqaaOGaemOzay2aa0baaSqaaiabdYgaSbqaamaabmaabaGaemOAaOgacaGLOaGaayzkaaaaaOGaey4kaSIae8xTdu2aa0baaSqaaiabdMgaPbqaamaabmaabaGaemOAaOgacaGLOaGaayzkaaaaaOGaeiilaWcaleaacqWGSbaBcqGH9aqpcqaIXaqmaeaacqWGmbata0GaeyyeIuoakiaaxMaacaWLjaWaaeWaaeaacqaIXaqmaiaawIcacaGLPaaaaaa@505E@

where f1(j),f2(j),…fL(j)
 MathType@MTEF@5@5@+=feaafiart1ev1aaatCvAUfKttLearuWrP9MDH5MBPbIqV92AaeXatLxBI9gBaebbnrfifHhDYfgasaacH8akY=wiFfYdH8Gipec8Eeeu0xXdbba9frFj0=OqFfea0dXdd9vqai=hGuQ8kuc9pgc9s8qqaq=dirpe0xb9q8qiLsFr0=vr0=vr0dc8meaabaqaciaacaGaaeqabaqabeGadaaakeaacqWGMbGzdaqhaaWcbaGaeGymaedabaWaaeWaaeaacqWGQbGAaiaawIcacaGLPaaaaaGccqGGSaalcqWGMbGzdaqhaaWcbaGaeGOmaidabaWaaeWaaeaacqWGQbGAaiaawIcacaGLPaaaaaGccqGGSaalcqWIMaYscqWGMbGzdaqhaaWcbaGaemitaWeabaWaaeWaaeaacqWGQbGAaiaawIcacaGLPaaaaaaaaa@3FDD@ are latent variables that affect the expression level. Others have applied latent variable models to analyze microarray data as well [13,14], although most of these studies use latent variable models in unsupervised ways and not directly to predict gene function.

In the absence of experimental covariates describing each experiment, we may treat each experiment as equivalent with both random noise and experimental differences modeled by the error part, εi(j)
 MathType@MTEF@5@5@+=feaafiart1ev1aaatCvAUfKttLearuWrP9MDH5MBPbIqV92AaeXatLxBI9gBaebbnrfifHhDYfgasaacH8akY=wiFfYdH8Gipec8Eeeu0xXdbba9frFj0=OqFfea0dXdd9vqai=hGuQ8kuc9pgc9s8qqaq=dirpe0xb9q8qiLsFr0=vr0=vr0dc8meaabaqaciaacaGaaeqabaqabeGadaaakeaaiiGacqWF1oqzdaqhaaWcbaGaemyAaKgabaWaaeWaaeaacqWGQbGAaiaawIcacaGLPaaaaaaaaa@32C8@. If experiments are controlled and experimental covariates are available, the above model may incorporate them by making *λ*_*il *_a function of covariates. This is one of the extensions to this model being currently studied by our group.

In matrix form, Eq. (1) can be written as:

**g**^(*j*) ^= **Λf**^(*j*) ^+ ***ε***^(*j*)^,     (2)

or

[g1(j)g2(j)⋮gp(j)]=[λ11λ12…λ1Lλ21λ22…λ2L⋮⋮⋱⋮λp1λp2⋯λpL][f1(j)f2(j)⋮fL(j)]+[ε1(j)ε2(j)⋮εp(j)].
 MathType@MTEF@5@5@+=feaafiart1ev1aaatCvAUfKttLearuWrP9MDH5MBPbIqV92AaeXatLxBI9gBaebbnrfifHhDYfgasaacH8akY=wiFfYdH8Gipec8Eeeu0xXdbba9frFj0=OqFfea0dXdd9vqai=hGuQ8kuc9pgc9s8qqaq=dirpe0xb9q8qiLsFr0=vr0=vr0dc8meaabaqaciaacaGaaeqabaqabeGadaaakeaadaWadaqaauaabeqaeeaaaaqaaiabdEgaNnaaDaaaleaacqaIXaqmaeaadaqadaqaaiabdQgaQbGaayjkaiaawMcaaaaaaOqaaiabdEgaNnaaDaaaleaacqaIYaGmaeaadaqadaqaaiabdQgaQbGaayjkaiaawMcaaaaaaOqaaiabl6UinbqaaiabdEgaNnaaDaaaleaacqWGWbaCaeaadaqadaqaaiabdQgaQbGaayjkaiaawMcaaaaaaaaakiaawUfacaGLDbaacqGH9aqpdaWadaqaauaabeqaeqaaaaaabaacciGae83UdW2aaSbaaSqaaiabigdaXiabigdaXaqabaaakeaacqWF7oaBdaWgaaWcbaGaeGymaeJaeGOmaidabeaaaOqaaiablAcilbqaaiab=T7aSnaaBaaaleaacqaIXaqmcqWGmbataeqaaaGcbaGae83UdW2aaSbaaSqaaiabikdaYiabigdaXaqabaaakeaacqWF7oaBdaWgaaWcbaGaeGOmaiJaeGOmaidabeaaaOqaaiablAcilbqaaiab=T7aSnaaBaaaleaacqaIYaGmcqWGmbataeqaaaGcbaGaeSO7I0eabaGaeSO7I0eabaGaeSy8I8eabaGaeSO7I0eabaGae83UdW2aaSbaaSqaaiabdchaWjabigdaXaqabaaakeaacqWF7oaBdaWgaaWcbaGaemiCaaNaeGOmaidabeaaaOqaaiabl+Uimbqaaiab=T7aSnaaBaaaleaacqWGWbaCcqWGmbataeqaaaaaaOGaay5waiaaw2faamaadmaabaqbaeqabqqaaaaabaGaemOzay2aa0baaSqaaiabigdaXaqaamaabmaabaGaemOAaOgacaGLOaGaayzkaaaaaaGcbaGaemOzay2aa0baaSqaaiabikdaYaqaamaabmaabaGaemOAaOgacaGLOaGaayzkaaaaaaGcbaGaeSO7I0eabaGaemOzay2aa0baaSqaaiabdYeambqaamaabmaabaGaemOAaOgacaGLOaGaayzkaaaaaaaaaOGaay5waiaaw2faaiabgUcaRmaadmaabaqbaeqabqqaaaaabaGae8xTdu2aa0baaSqaaiabigdaXaqaamaabmaabaGaemOAaOgacaGLOaGaayzkaaaaaaGcbaGae8xTdu2aa0baaSqaaiabikdaYaqaamaabmaabaGaemOAaOgacaGLOaGaayzkaaaaaaGcbaGaeSO7I0eabaGae8xTdu2aa0baaSqaaiabdchaWbqaamaabmaabaGaemOAaOgacaGLOaGaayzkaaaaaaaaaOGaay5waiaaw2faaiabc6caUaaa@9FD7@

Suppose Var(**g**) = **S**. In factor analysis, it is typically assumed that Var(**f**) = **I **is the identity matrix; Var(***ε***) = **E **= diag{σ12,σ22,…,σp2
 MathType@MTEF@5@5@+=feaafiart1ev1aaatCvAUfKttLearuWrP9MDH5MBPbIqV92AaeXatLxBI9gBaebbnrfifHhDYfgasaacH8akY=wiFfYdH8Gipec8Eeeu0xXdbba9frFj0=OqFfea0dXdd9vqai=hGuQ8kuc9pgc9s8qqaq=dirpe0xb9q8qiLsFr0=vr0=vr0dc8meaabaqaciaacaGaaeqabaqabeGadaaakeaaiiGacqWFdpWCdaqhaaWcbaGaeGymaedabaGaeGOmaidaaOGaeiilaWIae83Wdm3aa0baaSqaaiabikdaYaqaaiabikdaYaaakiabcYcaSiablAciljabcYcaSiab=n8aZnaaDaaaleaacqWGWbaCaeaacqaIYaGmaaaaaa@3C70@} is a diagonal matrix; and that ***ε ***and **f **are uncorrelated. We then obtain the following equation from (2):

Var(**g**) = **Λ**Var(**f**)**Λ**^*T *^+ Var(***ε***)

or

**S **= **ΛΛ**^*T *^+ **E**.     (3)

The matrix **S **can be estimated directly from the microarray data. Following general conventions in factor analysis, we take **S **to be the correlation matrix rather than the covariance matrix. This is equivalent to standardizing the data prior to the analysis and is customary in factor analysis [12].

#### A two-step algorithm

Our strategy for using this model to predict the gene functions is as follows: We first estimate **Λ **with Λ^
 MathType@MTEF@5@5@+=feaafiart1ev1aaatCvAUfKttLearuWrP9MDH5MBPbIqV92AaeXatLxBI9gBaebbnrfifHhDYfgasaacH8akY=wiFfYdH8Gipec8Eeeu0xXdbba9frFj0=OqFfea0dXdd9vqai=hGuQ8kuc9pgc9s8qqaq=dirpe0xb9q8qiLsFr0=vr0=vr0dc8meaabaqaciaacaGaaeqabaqabeGadaaakeaaiiqacuWFBoatgaqcaaaa@2E36@ using principal factor analysis. The number of latent variables, *L*, can be determined empirically by evaluating the goodness of Λ^
 MathType@MTEF@5@5@+=feaafiart1ev1aaatCvAUfKttLearuWrP9MDH5MBPbIqV92AaeXatLxBI9gBaebbnrfifHhDYfgasaacH8akY=wiFfYdH8Gipec8Eeeu0xXdbba9frFj0=OqFfea0dXdd9vqai=hGuQ8kuc9pgc9s8qqaq=dirpe0xb9q8qiLsFr0=vr0=vr0dc8meaabaqaciaacaGaaeqabaqabeGadaaakeaaiiqacuWFBoatgaqcaaaa@2E36@Λ^
 MathType@MTEF@5@5@+=feaafiart1ev1aaatCvAUfKttLearuWrP9MDH5MBPbIqV92AaeXatLxBI9gBaebbnrfifHhDYfgasaacH8akY=wiFfYdH8Gipec8Eeeu0xXdbba9frFj0=OqFfea0dXdd9vqai=hGuQ8kuc9pgc9s8qqaq=dirpe0xb9q8qiLsFr0=vr0=vr0dc8meaabaqaciaacaGaaeqabaqabeGadaaakeaaiiqacuWFBoatgaqcaaaa@2E36@^*T *^as an approximation to **S **[[12], p. 262–267], but we simply retain all factors with non-zero singular values.

Let Λ^
 MathType@MTEF@5@5@+=feaafiart1ev1aaatCvAUfKttLearuWrP9MDH5MBPbIqV92AaeXatLxBI9gBaebbnrfifHhDYfgasaacH8akY=wiFfYdH8Gipec8Eeeu0xXdbba9frFj0=OqFfea0dXdd9vqai=hGuQ8kuc9pgc9s8qqaq=dirpe0xb9q8qiLsFr0=vr0=vr0dc8meaabaqaciaacaGaaeqabaqabeGadaaakeaaiiqacuWFBoatgaqcaaaa@2E36@_1 _be the top *p*_1 _rows of Λ^
 MathType@MTEF@5@5@+=feaafiart1ev1aaatCvAUfKttLearuWrP9MDH5MBPbIqV92AaeXatLxBI9gBaebbnrfifHhDYfgasaacH8akY=wiFfYdH8Gipec8Eeeu0xXdbba9frFj0=OqFfea0dXdd9vqai=hGuQ8kuc9pgc9s8qqaq=dirpe0xb9q8qiLsFr0=vr0=vr0dc8meaabaqaciaacaGaaeqabaqabeGadaaakeaaiiqacuWFBoatgaqcaaaa@2E36@ corresponding to the characterized genes and let Λ^
 MathType@MTEF@5@5@+=feaafiart1ev1aaatCvAUfKttLearuWrP9MDH5MBPbIqV92AaeXatLxBI9gBaebbnrfifHhDYfgasaacH8akY=wiFfYdH8Gipec8Eeeu0xXdbba9frFj0=OqFfea0dXdd9vqai=hGuQ8kuc9pgc9s8qqaq=dirpe0xb9q8qiLsFr0=vr0=vr0dc8meaabaqaciaacaGaaeqabaqabeGadaaakeaaiiqacuWFBoatgaqcaaaa@2E36@_2 _be the bottom *p*_2 _rows of Λ^
 MathType@MTEF@5@5@+=feaafiart1ev1aaatCvAUfKttLearuWrP9MDH5MBPbIqV92AaeXatLxBI9gBaebbnrfifHhDYfgasaacH8akY=wiFfYdH8Gipec8Eeeu0xXdbba9frFj0=OqFfea0dXdd9vqai=hGuQ8kuc9pgc9s8qqaq=dirpe0xb9q8qiLsFr0=vr0=vr0dc8meaabaqaciaacaGaaeqabaqabeGadaaakeaaiiqacuWFBoatgaqcaaaa@2E36@ corresponding to the uncharacterized genes. We then derive a mapping from Λ^
 MathType@MTEF@5@5@+=feaafiart1ev1aaatCvAUfKttLearuWrP9MDH5MBPbIqV92AaeXatLxBI9gBaebbnrfifHhDYfgasaacH8akY=wiFfYdH8Gipec8Eeeu0xXdbba9frFj0=OqFfea0dXdd9vqai=hGuQ8kuc9pgc9s8qqaq=dirpe0xb9q8qiLsFr0=vr0=vr0dc8meaabaqaciaacaGaaeqabaqabeGadaaakeaaiiqacuWFBoatgaqcaaaa@2E36@_1 _to **Y**_1_, say *F*, and apply *F *to Λ^
 MathType@MTEF@5@5@+=feaafiart1ev1aaatCvAUfKttLearuWrP9MDH5MBPbIqV92AaeXatLxBI9gBaebbnrfifHhDYfgasaacH8akY=wiFfYdH8Gipec8Eeeu0xXdbba9frFj0=OqFfea0dXdd9vqai=hGuQ8kuc9pgc9s8qqaq=dirpe0xb9q8qiLsFr0=vr0=vr0dc8meaabaqaciaacaGaaeqabaqabeGadaaakeaaiiqacuWFBoatgaqcaaaa@2E36@_2_. We can picture the entire process as follows:

S→Step1Λ^=[Λ^1Λ^2]→Step2θ^=arg⁡min⁡ℓθ(F(Λ^1;θ),Y1)→PredictY^2=F(Λ^2;θ^).     (4)
 MathType@MTEF@5@5@+=feaafiart1ev1aaatCvAUfKttLearuWrP9MDH5MBPbIqV92AaeXatLxBI9gBaebbnrfifHhDYfgasaacH8akY=wiFfYdH8Gipec8Eeeu0xXdbba9frFj0=OqFfea0dXdd9vqai=hGuQ8kuc9pgc9s8qqaq=dirpe0xb9q8qiLsFr0=vr0=vr0dc8meaabaqaciaacaGaaeqabaqabeGadaaakeaaieqacqWFtbWucqWFGaaicqWFGaaifaqaaeqamaaaaaaabaWaa4ajaSqaaiabbofatjabbsha0jabbwgaLjabbchaWjabbgdaXaqabOGaayPKHaaabaacceGaf43MdWKbaKaaaeaacqGH9aqpaeaadaWadaqaauaabeqaceaaaeaacuGFBoatgaqcamaaBaaaleaacqaIXaqmaeqaaaGcbaGaf43MdWKbaKaadaWgaaWcbaGaeGOmaidabeaaaaaakiaawUfacaGLDbaaaeaadaGdKaWcbaGaee4uamLaeeiDaqNaeeyzauMaeeiCaaNaeeOmaidabeGccaGLsgcaaeaaiiWacuqF4oqCgaqcaaqaaGGaaiab81da9aqaamaaxababaGagiyyaeMaeiOCaiNaei4zaCMagiyBa0MaeiyAaKMaeiOBa4MaeS4eHWgaleaacqqF4oqCaeqaaOWaaeWaaeaacqWGgbGrdaqadaqaaiqb+T5amzaajaWaaSbaaSqaaiabigdaXaqabaGccqGG7aWocqqF4oqCaiaawIcacaGLPaaacqWFSaalcqWFzbqwdaWgaaWcbaGaeGymaedabeaaaOGaayjkaiaawMcaaaqaamaaoqcaleaacqqGqbaucqqGYbGCcqqGLbqzcqqGKbazcqqGPbqAcqqGJbWycqqG0baDaeqakiaawkziaaqaaiqb=LfazzaajaWaaSbaaSqaaiabikdaYaqabaaakeaacqGH9aqpaeaacqWGgbGrdaqadaqaaiqb+T5amzaajaWaaSbaaSqaaiabikdaYaqabaGccqGG7aWocuqF4oqCgaqcaaGaayjkaiaawMcaaiabc6caUaaacaWLjaGaaCzcamaabmaabaGaeGinaqdacaGLOaGaayzkaaaaaa@7EE2@

In this modeling paradigm, ℓ(·,·) denotes a loss function between the known associations **Y**_1 _and the predicted associations *F*(·;***θ***) based on the estimated loadings Λ^
 MathType@MTEF@5@5@+=feaafiart1ev1aaatCvAUfKttLearuWrP9MDH5MBPbIqV92AaeXatLxBI9gBaebbnrfifHhDYfgasaacH8akY=wiFfYdH8Gipec8Eeeu0xXdbba9frFj0=OqFfea0dXdd9vqai=hGuQ8kuc9pgc9s8qqaq=dirpe0xb9q8qiLsFr0=vr0=vr0dc8meaabaqaciaacaGaaeqabaqabeGadaaakeaaiiqacuWFBoatgaqcaaaa@2E36@_1_. The mapping *F*(·;***θ***) is assumed to be completely specified by a collection of parameters which we simply write as ***θ***, where ***θ ***is estimated by minimizing the loss function.

Details on the implementation of this two-step algorithm is given in the "Method" section below.

### Experiments

In this section, we discuss the set-up of our computational experiments, including descriptions of our data set, our preprocessing steps, and how performance is evaluated. We also briefly describe a benchmark SVM algorithm that we use for the purpose of comparison.

#### Data

We applied our model to the microarray data for yeast described in [1], but with functional annotations from the December 2004 release of the GO-BP table and the *Saccharomyces *Genome Database (SGD; also part of the GO consortium). The authors of [1] used a much simpler YPD classification scheme, which is not publicly available. Their microarray data contains 300 experiments from the Rosetta Compendium using genetic variants of yeast, as well as an additional publicly available data of 124 arrays from a set of three different experiments from other labs. Thus in total, there are 424 arrays with a common set of 6064 genes and ORFs. The full description of the data can be found in [1] and references therein.

#### Data preprocessing

We obtained the data from the authors of [1], where the data were already normalized and combined into one data matrix. Our further preprocessing steps included the following:

1. We deleted ORFs which are not listed in the newest (December 2004) SGD association table maintained by GO. Most of those had a "Dubious ORF" characterization in the SGD database.

2. We ignored the uncharacterized genes (and ORFs) – genes annotated to the node "Biological Process Unknown" in SGD.

3. We imputed missing values in the expression data using an adaptation of the k-nearest neighbor imputing algorithm for expression data described in [15]. We modified it so that the putative nearest neighbour gene profile, considered for imputing a given missing value, did not need to have a full expression profile as long as: (a) it had non-missing data in the column under consideration and (b) it had at least 80% expression values that were not missing. We used k = 10 nearest neighbours.

4. We restricted the set of GO-BP functional categories to those with at least 15 but no more than 100 genes associated to it. Since the goal is to produce functional predictions for genes to enable biologists to conduct more guided confirmatory experiments, we chose not to consider categories which had too few or too many genes associated to them. On the one hand, categories with too many genes annotated to them are less informative and hence not as interesting. On the other hand, categories with too few genes annotated to them cannot be credibly predicted.

5. Restricting GO categories produced a small list of "stranded" genes – genes which were otherwise known but whose annotations happened to be exclusively in the categories deleted from the list. We removed these "stranded" genes from our data set.

This resulted in 3224 genes and 369 functional categories. Specifically, we formed a 3224 × 424 expression data matrix **Z **(see [1]) and a 3224 × 369 association (response) matrix **Y **[see Additional file 1].

#### Benchmark

To compare the performance of our method, we also applied the popular approach in functional genomics: function-wise binary classification using Support Vector Machines (SVMs). SVM is a well-known method in machine learning (see [16] for links to much of the literature, tutorials and software) and is used by [3] to classify mouse genes.

We chose Gist 2.1.1 [17] as our SVM software due to its tailored interface for microarray data and because of its use in [3]. We tested several different values via cross-validation for the following tuning parameters in SVM: the type of kernel (linear, polynomial, and radial basis functions), the misclassification penalty (diagfactor in Gist), and the width factor (widthfactor in Gist) that controls the bandwidth of the radial basis kernel, which is calculated as (width) × (width factor). The "width" is taken to be the median distance from a data point to its nearest neighbor of the opposite class; the "width factor" is specified by the user. Further details on the parameter selection process are elaborated in the "Methods" section.

#### Performance evaluation

Although classifiers are typically evaluated by the misclassification rate in the statistics and machine learning literature, it is *not *a useful criterion for functional genomics from a biologist's point of view. Since the most interesting functional categories are those that are fairly rare, the vast majority of genes would *not *be associated with such a category. This means that even poor classifiers can achieve very low misclassification rates by simply predicting that no genes are associated with that category (i.e. by classifying everything to the majority class). Instead, suppose the prediction algorithm ranks genes according to predicted likelihood of association to that category (e.g., FAM can rank genes using estimated posterior probabilities via naïve Bayes – see the "Method" section). Biologists are most interested in using a computational algorithm to generate a list of candidate genes which can be further investigated in the lab. Since such investigations are often labourious and costly, a highly relevant measure of success should determine whether the algorithm ranks the truly associated genes ahead of the unassociated ones.

In the biomedical literature, prediction accuracy is often evaluated by *sensitivity *and *specificity *[18]. Suppose we focus on *m *<*p *top-ranked genes. Sensitivity is the probability that a gene appears in this top-ranked list given that it is truly associated, whereas specificity is the probability that a gene does not appear in this list given that it is not associated. They both depend on the number *m*, the size of the top-ranked list. The 2 × 2 Table 1 shows how these quantities can be calculated. Hence, we have:

sensitivity=aa+b=asandspecificity=dc+d=p−m−bp−s.
 MathType@MTEF@5@5@+=feaafiart1ev1aaatCvAUfKttLearuWrP9MDH5MBPbIqV92AaeXatLxBI9gBaebbnrfifHhDYfgasaacH8akY=wiFfYdH8Gipec8Eeeu0xXdbba9frFj0=OqFfea0dXdd9vqai=hGuQ8kuc9pgc9s8qqaq=dirpe0xb9q8qiLsFr0=vr0=vr0dc8meaabaqaciaacaGaaeqabaqabeGadaaakeaafaqabeqadaaabaGaee4CamNaeeyzauMaeeOBa4Maee4CamNaeeyAaKMaeeiDaqNaeeyAaKMaeeODayNaeeyAaKMaeeiDaqNaeeyEaKNaeyypa0ZaaSaaaeaacqWGHbqyaeaacqWGHbqycqGHRaWkcqWGIbGyaaGaeyypa0ZaaSaaaeaacqWGHbqyaeaacqWGZbWCaaaabaGaeeyyaeMaeeOBa4MaeeizaqgabaGaee4CamNaeeiCaaNaeeyzauMaee4yamMaeeyAaKMaeeOzayMaeeyAaKMaee4yamMaeeyAaKMaeeiDaqNaeeyEaKhaaiabg2da9maalaaabaGaemizaqgabaGaem4yamMaey4kaSIaemizaqgaaiabg2da9maalaaabaGaemiCaaNaeyOeI0IaemyBa0MaeyOeI0IaemOyaigabaGaemiCaaNaeyOeI0Iaem4Camhaaiabc6caUaaa@6A64@

The number *m *is typically determined by external factors such as how much funding there is to perform confirmatory experiments. Full confirmation or rejection of a putative association predicted from a genome-wide scan will typically involve classical experiments from molecular biology or biochemistry over a longer time span. A given lab may decide to pursue a few of these candidate genes, taking into account their ranking from prediction tools as well as additional information such as specific reagents needed and lab's area of expertise. However an intermediate screening step which we have in mind, such as quantitative RT-PCR, may be performed on a larger subset of candidates.

In order to compare two prediction algorithms, one needs to decide on the threshold *m*. Objectively derived statistical thresholds are hard to estimate and do not always reflect the reality of the biologist. We decided on a fixed threshold of *m *such that the top-ranked list consists of 10% of the genes, which we felt was a realistic compromise. For example, yeast has about 2000 uncharacterized genes, and attempting to furtherscreen the top 200 of them by lower-throughput methods such as quantitative RT-PCR would be feasible in a typical lab.

For predicting rare functional categories, sensitivity is more important than specificity. Again, refer to Table 1. By definition, *s *is small compared to *p *for rare functional categories. Clearly, *b *<*s*. Therefore,

specificity=dc+d=p−m−bp−s≈p−mp,
 MathType@MTEF@5@5@+=feaafiart1ev1aaatCvAUfKttLearuWrP9MDH5MBPbIqV92AaeXatLxBI9gBaebbnrfifHhDYfgasaacH8akY=wiFfYdH8Gipec8Eeeu0xXdbba9frFj0=OqFfea0dXdd9vqai=hGuQ8kuc9pgc9s8qqaq=dirpe0xb9q8qiLsFr0=vr0=vr0dc8meaabaqaciaacaGaaeqabaqabeGadaaakeaacqqGZbWCcqqGWbaCcqqGLbqzcqqGJbWycqqGPbqAcqqGMbGzcqqGPbqAcqqGJbWycqqGPbqAcqqG0baDcqqG5bqEcqGH9aqpdaWcaaqaaiabdsgaKbqaaiabdogaJjabgUcaRiabdsgaKbaacqGH9aqpdaWcaaqaaiabdchaWjabgkHiTiabd2gaTjabgkHiTiabdkgaIbqaaiabdchaWjabgkHiTiabdohaZbaacqGHijYUdaWcaaqaaiabdchaWjabgkHiTiabd2gaTbqaaiabdchaWbaacqGGSaalaaa@542B@

which is roughly constant for fixed *m*. This means all algorithms will have very similar specificity values.

In practice, the choice of *m *must reflect an acceptable trade-off between falsely detecting an unassociated gene and failing to detect an associated gene. Given that our choice of *m *is somewhat arbitrary, it is important for us to consider a more global performance measure. According to [18], the receiver operating characteristic (ROC) curve is currently the best-developed statistical tool for evaluating ranking algorithms; it "describes the [entire] range of trade-offs that can be achieved" by a given algorithm.

The area under the ROC curve – often referred to simply as AUC in the biomedical literature – is a widely used summary measure of the ROC curve. Let *g *and g¯
 MathType@MTEF@5@5@+=feaafiart1ev1aaatCvAUfKttLearuWrP9MDH5MBPbIqV92AaeXatLxBI9gBaebbnrfifHhDYfgasaacH8akY=wiFfYdH8Gipec8Eeeu0xXdbba9frFj0=OqFfea0dXdd9vqai=hGuQ8kuc9pgc9s8qqaq=dirpe0xb9q8qiLsFr0=vr0=vr0dc8meaabaqaciaacaGaaeqabaqabeGadaaakeaacuWGNbWzgaqeaaaa@2E1B@ be a randomly selected pair of associated and unassociated genes, and *r*_*g *_and rg¯
 MathType@MTEF@5@5@+=feaafiart1ev1aaatCvAUfKttLearuWrP9MDH5MBPbIqV92AaeXatLxBI9gBaebbnrfifHhDYfgasaacH8akY=wiFfYdH8Gipec8Eeeu0xXdbba9frFj0=OqFfea0dXdd9vqai=hGuQ8kuc9pgc9s8qqaq=dirpe0xb9q8qiLsFr0=vr0=vr0dc8meaabaqaciaacaGaaeqabaqabeGadaaakeaacqWGYbGCdaWgaaWcbaGafm4zaCMbaebaaeqaaaaa@2FB4@ be their respective rankings. It is well-known [18,19] that AUC is equal to the probability that *g *and g¯
 MathType@MTEF@5@5@+=feaafiart1ev1aaatCvAUfKttLearuWrP9MDH5MBPbIqV92AaeXatLxBI9gBaebbnrfifHhDYfgasaacH8akY=wiFfYdH8Gipec8Eeeu0xXdbba9frFj0=OqFfea0dXdd9vqai=hGuQ8kuc9pgc9s8qqaq=dirpe0xb9q8qiLsFr0=vr0=vr0dc8meaabaqaciaacaGaaeqabaqabeGadaaakeaacuWGNbWzgaqeaaaa@2E1B@ are correctly ordered by the ranking algorithm, i.e., AUC = *P*(*r*_*g *_<rg¯
 MathType@MTEF@5@5@+=feaafiart1ev1aaatCvAUfKttLearuWrP9MDH5MBPbIqV92AaeXatLxBI9gBaebbnrfifHhDYfgasaacH8akY=wiFfYdH8Gipec8Eeeu0xXdbba9frFj0=OqFfea0dXdd9vqai=hGuQ8kuc9pgc9s8qqaq=dirpe0xb9q8qiLsFr0=vr0=vr0dc8meaabaqaciaacaGaaeqabaqabeGadaaakeaacqWGYbGCdaWgaaWcbaGafm4zaCMbaebaaeqaaaaa@2FB4@). Therefore, AUC is a good (and threshold-free) performance measure for determining whether an algorithm can rank truly associated genes ahead of the unassociated ones. The use of AUC as a performance criterion is also becoming more popular in the machine learning community [20]. As the averaged results later illustrate, SVM and FAM tend to perform comparably on the AUC scale.

### Computational results

We validated our method with a four-fold cross-validation (C-V) experiment. We used four folds (rather than ten folds, for example) because of the need to retain a reasonable number of associated genes in each fold for each functional category, which we verified *post hoc*. It is important to note that the C-V folds were obtained by an unconstrained randomization process.

More specifically, the 3224 genes were randomly divided into 4 groups of 806 each. In each C-V run, one of the four folds is designated as the test set and the corresponding part of the association matrix, **Y**, removed. The remaining three quarters of the data were designated as the training set. Then both FAM and SVM were fitted to the training data and then applied to the test data to obtain predictions. For FAM, the prediction was based on the estimated posterior probability (see Method section for more details); for SVM, the prediction was based on the signed distance to the separating hyperplane. The test genes would then be ranked by each method for each function separately and the three performance metrics (sensitivity and specificity for the 10% top-ranked genes, and area underneath the ROC curve) calculated.

#### Computational details

FAM is implemented in R [21]. Executing the four-fold C-V experiment, run serially across the 369 functional categories, took less than 5 CPU minutes on a dual-CPU (3 GHz Pentium) Linux desktop with 2 GB of RAM. Gist is implemented in ANSI C. Executing the four-fold C-V experiment, run in *parallel *across the 369 functional categories, took at least 12 hours on a 32 CPU (l.3 GHz Itanium II each) SGI Altix server with 64 GB of RAM (where each of the processors were fully loaded for the 12 hours). This corresponds to approximately 23040 CPU minutes (12 hours × 60 × 32 CPUs). Thus, compared to FAM's running time of 5 CPU minutes, training the SVMs took approximately 4000 times longer.

#### Prediction results

Tables 2 and 3 show the main results from our four-fold C-V experiments. Table 2 compares the performance of FAM and SVM, averaged across all 369 functional categories. Table 3 makes pairwise comparisons of the predictions made by FAM and SVM for the 369 GO functions in the four C-V experiments. For each of the 369 GO functions, the performance of FAM and SVM are compared, and Table 3 reports the number of GO functions for which FAM's predictions are better than, tied with, or worse than that of SVM. The last row, denoted by "ACV," compares FAM and SVM using averaged performance metrics over the four C-V folds, which give rise to much fewer ties.

The general conclusion is that the two methods are very similar in terms of their predictive performances (as measured by the three performance metrics). FAM is slightly better than SVM based on the ACV pairwise comparison (Table 3). These differences are not statistically significant, but we are greatly encouraged by these results given the tremendous computational advantages of FAM.

Tables 4 and 5 are similar to Tables 2 and 3, except the 369 functional categories are partitioned into 4 groups based on how informative they are. We say that a GO function is more informative if fewer genes are associatedwith it. The first group (H) consists of categories with only 15 to 20 associated genes (out of a total of 3224) – these functional categories are highly informative; there are 95 such categories. The second and third group consist, respectively, of categories with 21 to 40, and 41 to 60 associated genes, which we label as Medium High (MH) and Medium Low (ML) on the informativeness scale. There are 144 and 71 such functional categories in these two groups, respectively. The least informative group (L) contains functions with over 60 associated genes; there are 59 such GO categories.

**Figure 1:**
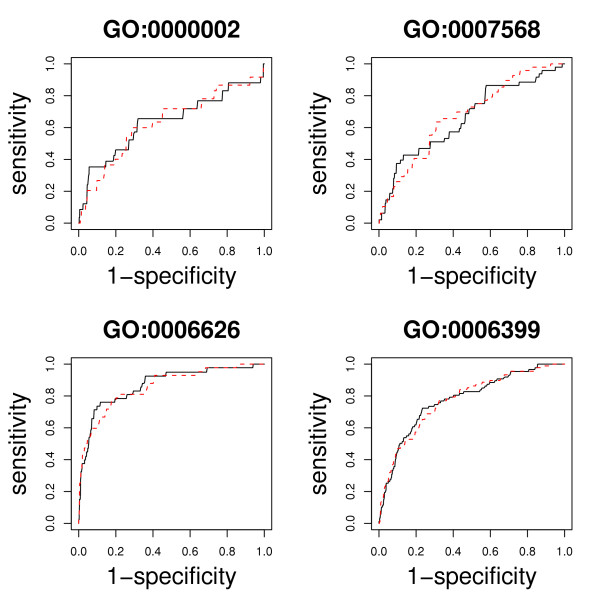
**four sample ROC curves. **We group 369 GO functions into four groups based on how informative they are. We say a GO function is more informative if fewer genes are associated with it. H, MH, ML, L denote the 4 groups with high, medium-high, medium-low, and low levels of informativeness. Here, we show sample ROC curves for four randomly selected GO functions, one from each of the four groups. G0:0000002 = "mitochondrial genome maintenance" (from group H); G0:0007568 = "aging" (from group MH); G0:0006626 = "protein targeting to mitochondrion" (from group ML); and G0:0006399 = "tRNA metabolism" (from group L). Solid line = FAM; dashed line = SVM.

To illustrate, we randomly choose one GO function from each of the four groups described above (H, MH, ML, and L) and display the corresponding sample ROC curves of the two methods in Figure 1. The curves produced by FAM and SVM are quite similar; naturally, we would expect them to have similar areas underneath as well. We also observe that the ROC curves of FAM tend to slightly dominate in the early part, where specificity is high. This explains why FAM tends to perform slightly better in terms of sensitivity at low thresholds (see Table 2), although the differences here are quite small. Full prediction results for FAM and SVM are in [Additional file 2] and [Additional file 3], respectively.

## Discussion

In terms of using microarray expression data to identify uncharacterized genes that may be associated with various biological functions, FAM appearsto be as effective as SVM, a powerful machine learning tool. However, our FAM-based algorithm is faster than the SVM by a factor of 4000.

More importantly, the FAM framework has a great potential for incorporating important gene ontology information and dealing with a number of major challenges in using statistical classification techniques for functional genomics.

### FAM as a functional random-effects model

The hierarchical nature of how the functional categories are organized in GO-BP imply that some of these functional categories are highly correlated. A very promising and important extension of FAM is the possibility of taking this correlation structure into account. More specifically, with a total of *K *different functional categories, we can model the expression level gi(j)
 MathType@MTEF@5@5@+=feaafiart1ev1aaatCvAUfKttLearuWrP9MDH5MBPbIqV92AaeXatLxBI9gBaebbnrfifHhDYfgasaacH8akY=wiFfYdH8Gipec8Eeeu0xXdbba9frFj0=OqFfea0dXdd9vqai=hGuQ8kuc9pgc9s8qqaq=dirpe0xb9q8qiLsFr0=vr0=vr0dc8meaabaqaciaacaGaaeqabaqabeGadaaakeaacqWGNbWzdaqhaaWcbaGaemyAaKgabaWaaeWaaeaacqWGQbGAaiaawIcacaGLPaaaaaaaaa@3271@ explicitly as being dependent on the *K *functional categories, i.e.,

gi(j)=∑k=1Kλikfk(j)+εi(j),     (5)
 MathType@MTEF@5@5@+=feaafiart1ev1aaatCvAUfKttLearuWrP9MDH5MBPbIqV92AaeXatLxBI9gBaebbnrfifHhDYfgasaacH8akY=wiFfYdH8Gipec8Eeeu0xXdbba9frFj0=OqFfea0dXdd9vqai=hGuQ8kuc9pgc9s8qqaq=dirpe0xb9q8qiLsFr0=vr0=vr0dc8meaabaqaciaacaGaaeqabaqabeGadaaakeaacqWGNbWzdaqhaaWcbaGaemyAaKgabaWaaeWaaeaacqWGQbGAaiaawIcacaGLPaaaaaGccqGH9aqpdaaeWbqaaGGaciab=T7aSnaaBaaaleaacqWGPbqAcqWGRbWAaeqaaOGaemOzay2aa0baaSqaaiabdUgaRbqaamaabmaabaGaemOAaOgacaGLOaGaayzkaaaaaOGaey4kaSIae8xTdu2aa0baaSqaaiabdMgaPbqaamaabmaabaGaemOAaOgacaGLOaGaayzkaaaaaOGaeiilaWIaaCzcaiaaxMaadaqadaqaaiabiwda1aGaayjkaiaawMcaaaWcbaGaem4AaSMaeyypa0JaeGymaedabaGaem4saSeaniabggHiLdaaaa@5054@

where fk(j)
 MathType@MTEF@5@5@+=feaafiart1ev1aaatCvAUfKttLearuWrP9MDH5MBPbIqV92AaeXatLxBI9gBaebbnrfifHhDYfgasaacH8akY=wiFfYdH8Gipec8Eeeu0xXdbba9frFj0=OqFfea0dXdd9vqai=hGuQ8kuc9pgc9s8qqaq=dirpe0xb9q8qiLsFr0=vr0=vr0dc8meaabaqaciaacaGaaeqabaqabeGadaaakeaacqWGMbGzdaqhaaWcbaGaem4AaSgabaWaaeWaaeaacqWGQbGAaiaawIcacaGLPaaaaaaaaa@3273@ is now a (non-latent) random effect for function *k*. Thus, there are two random components in this model: the function-dependent random factors, fk(j)
 MathType@MTEF@5@5@+=feaafiart1ev1aaatCvAUfKttLearuWrP9MDH5MBPbIqV92AaeXatLxBI9gBaebbnrfifHhDYfgasaacH8akY=wiFfYdH8Gipec8Eeeu0xXdbba9frFj0=OqFfea0dXdd9vqai=hGuQ8kuc9pgc9s8qqaq=dirpe0xb9q8qiLsFr0=vr0=vr0dc8meaabaqaciaacaGaaeqabaqabeGadaaakeaacqWGMbGzdaqhaaWcbaGaem4AaSgabaWaaeWaaeaacqWGQbGAaiaawIcacaGLPaaaaaaaaa@3273@, realized independently for each microarray experiment *j*, and the error term, εi(j)
 MathType@MTEF@5@5@+=feaafiart1ev1aaatCvAUfKttLearuWrP9MDH5MBPbIqV92AaeXatLxBI9gBaebbnrfifHhDYfgasaacH8akY=wiFfYdH8Gipec8Eeeu0xXdbba9frFj0=OqFfea0dXdd9vqai=hGuQ8kuc9pgc9s8qqaq=dirpe0xb9q8qiLsFr0=vr0=vr0dc8meaabaqaciaacaGaaeqabaqabeGadaaakeaaiiGacqWF1oqzdaqhaaWcbaGaemyAaKgabaWaaeWaaeaacqWGQbGAaiaawIcacaGLPaaaaaaaaa@32C8@, which is realized independently for each microarray (*j*) and each gene (*i*).

As stated above, these (functional) random effects can no longer be assumed to be independent. Let **Φ **≡ Var(**f**) ≠ **I **be the covariance matrix of these random effects. Eq. (3) then becomes

S = **ΛΦΛ**^*T *^+ **E**.     (6)

When properly estimated, the number *λ*_*ik *_in this model can be interpreted as the "coefficient of association" between gene *i *and function *k *and used directly to predict gene functions.

Hence, FAM has the potential to explicitly account for the fact that both the gene expression profiles and the functional categories are correlated. By contrast, applying a classifier such as SVM separately for each functional category implicitly assumes that both are independent.

The challenge lies in the estimation of the covariance matrix **Φ **based on the DAG structure of GO-BP. This is a non-trivial but interesting challenge. So far, we have experimented with three different methods to obtain a *similarity *matrix: Lin similarity [22], which has previously been applied in microarray data [7]; Resnik similarity [23], which has also been applied to microarray data [24]; and a flexible Jiang and Conrath similarity [25], which has been extended to specifically apply to estimate gene-gene similarity using GO-BP by [26]. Such similarity matrices then need to be transformed to a valid (i.e., positive-definite) covariance matrix, which can be used as an estimate of **Φ**. After experimenting with these different techniques, we obtained very different estimates of **Φ**. At the current state, it is difficult to evaluate which estimate is better than others. We will report more details in our future work.

### FAM with functional constraints

If it is known that there is no association between gene *i *and function *k*, we can set *λ*_*ik *_= 0 in Eq. (6) and estimate the remaining entries of **Λ **from data. This is closely related to *confirmatory factor analysis *[27]. Using the notation above, we wish to find values for the unknown entries of the matrix **Λ **that optimally "fits" Eq. (6). For example, we can use the Frobenious norm as the loss function and minimize the function:

||**S **- **ΛΦΛ**^*T *^- **E**||_*F*_.     (7)

If *λ*_*ik *_are restricted to be 0 or 1, this problem would be a nonlinear binary optimization problem, which is computationally taxing but potentially solvable due to recent breakthroughs in integer programming [28]. The continuous relaxation (when the restrictions *λ*_*ik *_= 0 or 1 are removed) is still a non-trivial problem since the objective function is non-convex with respect to **Λ **[29]. Nevertheless, we explored various techniques to tackle non-convex integer programming problems, including approximating the solution by a semi-definite programming formulation (SDP) – a technique that has been successful in many non-convex optimization problems [30]. Although the Lagrangian relaxation of minimizing (7) can be modeled as an SDP problem [30], the objective value is often non-positive. Thus, this relaxation did not lead to a usable solution.

There has been some recent advancements in the area of non-convex integer programming problems, where researchers from chemical engineering and optimization have joined forces to develop a practical software [31]. In the future, improvements in computation time of these algorithms may allow us to solve our problem to global optimality.

### Other extensions

Finally, even by considering just the basic FAM framework (3) alone leads to a number of different algorithms. For example, we are currently exploring alternatives to principal factor analysis such as maximum likelihood or Bayesian factor analysis. As mentioned earlier, our FAM-based algorithm has a big computational advantage. Alternatives mentioned such as maximum likelihood and Bayesian factor analysis are computationally more demanding but we expect that they would retain their computational advantage over training hundreds of SVMs to a data set comprised of thousands of gene expression vectors.

## Conclusion

We now summarize the main contributions of our work. First, we proposed a model and an algorithm based on factor analysis (FAM) for predicting gene functions using microarray expression data. We evaluated the performance of our algorithm with data previously analyzed by [1]. By running a four-fold cross-validation experiment using only characterized genes, we showed that our method is not only orders of magnitude faster, but performs comparably against a sophisticated, state-of-the-art classifier that is being widely used in functional genomics. Second, we showed that our FAM framework will allow us to incorporate important gene ontology information and hence deal with a number of major challenges in using statistical classification techniques for functional genomics. Most importantly, it will allow us to directly account for the fact that the functional categories are inherently correlated and exploit this correlation structure for prediction.

## Method

This section elaborates on the details of our implementation both for FAM and SVM.

### Algorithm implementation

Here, we give more details for how we implemented the two-step algorithm based on our factor analysis model.

#### Step 1: Principal factor analysis

To estimate **Λ**, we choose Λ^
 MathType@MTEF@5@5@+=feaafiart1ev1aaatCvAUfKttLearuWrP9MDH5MBPbIqV92AaeXatLxBI9gBaebbnrfifHhDYfgasaacH8akY=wiFfYdH8Gipec8Eeeu0xXdbba9frFj0=OqFfea0dXdd9vqai=hGuQ8kuc9pgc9s8qqaq=dirpe0xb9q8qiLsFr0=vr0=vr0dc8meaabaqaciaacaGaaeqabaqabeGadaaakeaaiiqacuWFBoatgaqcaaaa@2E36@ and E^
 MathType@MTEF@5@5@+=feaafiart1ev1aaatCvAUfKttLearuWrP9MDH5MBPbIqV92AaeXatLxBI9gBaebbnrfifHhDYfgasaacH8akY=wiFfYdH8Gipec8Eeeu0xXdbba9frFj0=OqFfea0dXdd9vqai=hGuQ8kuc9pgc9s8qqaq=dirpe0xb9q8qiLsFr0=vr0=vr0dc8meaabaqaciaacaGaaeqabaqabeGadaaakeaaieqacuWFfbqrgaqcaaaa@2DD5@ that minimize some loss criterion ℓ(**S**, **ΛΛ **+ **E**). For example, one may choose ℓ(**A**, **B**) to be the Frobenious norm of the difference **A **- **B **and choose Λ^
 MathType@MTEF@5@5@+=feaafiart1ev1aaatCvAUfKttLearuWrP9MDH5MBPbIqV92AaeXatLxBI9gBaebbnrfifHhDYfgasaacH8akY=wiFfYdH8Gipec8Eeeu0xXdbba9frFj0=OqFfea0dXdd9vqai=hGuQ8kuc9pgc9s8qqaq=dirpe0xb9q8qiLsFr0=vr0=vr0dc8meaabaqaciaacaGaaeqabaqabeGadaaakeaaiiqacuWFBoatgaqcaaaa@2E36@ and E^
 MathType@MTEF@5@5@+=feaafiart1ev1aaatCvAUfKttLearuWrP9MDH5MBPbIqV92AaeXatLxBI9gBaebbnrfifHhDYfgasaacH8akY=wiFfYdH8Gipec8Eeeu0xXdbba9frFj0=OqFfea0dXdd9vqai=hGuQ8kuc9pgc9s8qqaq=dirpe0xb9q8qiLsFr0=vr0=vr0dc8meaabaqaciaacaGaaeqabaqabeGadaaakeaaieqacuWFfbqrgaqcaaaa@2DD5@ that solve:

min⁡Λ,Ε‖S−(ΛΛT+E)‖F.     (8)
 MathType@MTEF@5@5@+=feaafiart1ev1aaatCvAUfKttLearuWrP9MDH5MBPbIqV92AaeXatLxBI9gBaebbnrfifHhDYfgasaacH8akY=wiFfYdH8Gipec8Eeeu0xXdbba9frFj0=OqFfea0dXdd9vqai=hGuQ8kuc9pgc9s8qqaq=dirpe0xb9q8qiLsFr0=vr0=vr0dc8meaabaqaciaacaGaaeqabaqabeGadaaakeaadaWfqaqaaiGbc2gaTjabcMgaPjabc6gaUbWcbaacceGae83MdWKae8hlaWIae8xLdueabeaakmaafmaabaacbeGae43uamLaeyOeI0YaaeWaaeaacqWFBoatcqWFBoatdaahaaWcbeqaaiabdsfaubaakiabgUcaRiab+veafbGaayjkaiaawMcaaaGaayzcSlaawQa7amaaBaaaleaacqWGgbGraeqaaOGaeiOla4IaaCzcaiaaxMaadaqadaqaaiabiIda4aGaayjkaiaawMcaaaaa@47CB@

In our procedure, we use the most popular algorithm for fitting a factor analysis model known as Principal Factor Analysis [[12], p. 261], but one could also use maximum likelihood or Bayesian factor analysis to estimate these quantities (see the "Discussion" section). Principal factor analysis produces an approximate solution, Λ^
 MathType@MTEF@5@5@+=feaafiart1ev1aaatCvAUfKttLearuWrP9MDH5MBPbIqV92AaeXatLxBI9gBaebbnrfifHhDYfgasaacH8akY=wiFfYdH8Gipec8Eeeu0xXdbba9frFj0=OqFfea0dXdd9vqai=hGuQ8kuc9pgc9s8qqaq=dirpe0xb9q8qiLsFr0=vr0=vr0dc8meaabaqaciaacaGaaeqabaqabeGadaaakeaaiiqacuWFBoatgaqcaaaa@2E36@, to Eq. (8), where the columns of Λ^
 MathType@MTEF@5@5@+=feaafiart1ev1aaatCvAUfKttLearuWrP9MDH5MBPbIqV92AaeXatLxBI9gBaebbnrfifHhDYfgasaacH8akY=wiFfYdH8Gipec8Eeeu0xXdbba9frFj0=OqFfea0dXdd9vqai=hGuQ8kuc9pgc9s8qqaq=dirpe0xb9q8qiLsFr0=vr0=vr0dc8meaabaqaciaacaGaaeqabaqabeGadaaakeaaiiqacuWFBoatgaqcaaaa@2E36@ are uncorrelated.

#### Step 2: Naïve Bayes

The fact that the columns of Λ^
 MathType@MTEF@5@5@+=feaafiart1ev1aaatCvAUfKttLearuWrP9MDH5MBPbIqV92AaeXatLxBI9gBaebbnrfifHhDYfgasaacH8akY=wiFfYdH8Gipec8Eeeu0xXdbba9frFj0=OqFfea0dXdd9vqai=hGuQ8kuc9pgc9s8qqaq=dirpe0xb9q8qiLsFr0=vr0=vr0dc8meaabaqaciaacaGaaeqabaqabeGadaaakeaaiiqacuWFBoatgaqcaaaa@2E36@ are uncorrelated allows us to use very simple procedures to construct the mapping *F*, e.g., the naïve Bayes method [[32], Section 6.6.3], which fully exploits the uncorrelated nature of the inputs. In order to apply naïve Bayes, we break the mapping *F *into *L *components, *F *= {*F*_*l*_; *l *= 1, 2, ..., *L*}; each *F*_*l *_uses the naïve Bayes method to map Λ^
 MathType@MTEF@5@5@+=feaafiart1ev1aaatCvAUfKttLearuWrP9MDH5MBPbIqV92AaeXatLxBI9gBaebbnrfifHhDYfgasaacH8akY=wiFfYdH8Gipec8Eeeu0xXdbba9frFj0=OqFfea0dXdd9vqai=hGuQ8kuc9pgc9s8qqaq=dirpe0xb9q8qiLsFr0=vr0=vr0dc8meaabaqaciaacaGaaeqabaqabeGadaaakeaaiiqacuWFBoatgaqcaaaa@2E36@ to the *l*^*th *^column of **Y**.

#### The Naïve Bayes method

Let *y *∈ {0,1} be the class label and **x **∈ ℝ^*L*^, the vector of predictors. Suppose *p*_0_(**x**) and *p*_1_(**x**) are the density functions for the two classes. The posterior probability that an observation belongs to class 1 is simply

P(y=1|x)=π1p1(x)π1p1(x)+π0p0(x)     (9)
 MathType@MTEF@5@5@+=feaafiart1ev1aaatCvAUfKttLearuWrP9MDH5MBPbIqV92AaeXatLxBI9gBaebbnrfifHhDYfgasaacH8akY=wiFfYdH8Gipec8Eeeu0xXdbba9frFj0=OqFfea0dXdd9vqai=hGuQ8kuc9pgc9s8qqaq=dirpe0xb9q8qiLsFr0=vr0=vr0dc8meaabaqaciaacaGaaeqabaqabeGadaaakeaacqWGqbaudaqadaqaaiabdMha5jabg2da9iabigdaXiabcYha8Hqabiab=Hha4bGaayjkaiaawMcaaiabg2da9maalaaabaacciGae4hWda3aaSbaaSqaaiabigdaXaqabaGccqWGWbaCdaWgaaWcbaGaeGymaedabeaakmaabmaabaGae8hEaGhacaGLOaGaayzkaaaabaGae4hWda3aaSbaaSqaaiabigdaXaqabaGccqWGWbaCdaWgaaWcbaGaeGymaedabeaakmaabmaabaGae8hEaGhacaGLOaGaayzkaaGaey4kaSIae4hWda3aaSbaaSqaaiabicdaWaqabaGccqWGWbaCdaWgaaWcbaGaeGimaadabeaakmaabmaabaGae8hEaGhacaGLOaGaayzkaaaaaiaaxMaacaWLjaWaaeWaaeaacqaI5aqoaiaawIcacaGLPaaaaaa@54D9@

where *π*_1 _and *π*_0 _are prior probabilities. The naive Bayes method simply assumes the density functions *p*_1 _and *p*_0 _to be of a product form:

p0(x)=∏l=1Lp0,l(xl)andp1(x)=∏l=1Lp1,l(xl).
 MathType@MTEF@5@5@+=feaafiart1ev1aaatCvAUfKttLearuWrP9MDH5MBPbIqV92AaeXatLxBI9gBaebbnrfifHhDYfgasaacH8akY=wiFfYdH8Gipec8Eeeu0xXdbba9frFj0=OqFfea0dXdd9vqai=hGuQ8kuc9pgc9s8qqaq=dirpe0xb9q8qiLsFr0=vr0=vr0dc8meaabaqaciaacaGaaeqabaqabeGadaaakeaafaqabeqadaaabaGaemiCaa3aaSbaaSqaaiabicdaWaqabaGcdaqadaqaaGqabiab=Hha4bGaayjkaiaawMcaaiabg2da9maarahabaGaemiCaa3aaSbaaSqaaiabicdaWiabcYcaSiabdYgaSbqabaGcdaqadaqaaiabdIha4naaBaaaleaacqWGSbaBaeqaaaGccaGLOaGaayzkaaaaleaacqWGSbaBcqGH9aqpcqaIXaqmaeaacqWGmbata0Gaey4dIunaaOqaaiabbggaHjabb6gaUjabbsgaKbqaaiabdchaWnaaBaaaleaacqaIXaqmaeqaaOWaaeWaaeaacqWF4baEaiaawIcacaGLPaaacqGH9aqpaaWaaebCaeaacqWGWbaCdaWgaaWcbaGaeGymaeJaeiilaWIaemiBaWgabeaakmaabmaabaGaemiEaG3aaSbaaSqaaiabdYgaSbqabaaakiaawIcacaGLPaaaaSqaaiabdYgaSjabg2da9iabigdaXaqaaiabdYeambqdcqGHpis1aOGaeiOla4caaa@5EFE@

In other words, the elements of the input vector **x **are assumed to be independent. The marginal density functions *p*_0,*l *_and *p*_1,*l *_can be estimated with standard (univariate) density estimators.

For any given functional category, often only a few genes are associated with it, meaning that class 1 is relatively rare. Under such circumstances, it is often necessary to impose additional parametric assumptions on the density functions before they can be estimated. We assume (as is also commonly done) that both *p*_0,*l *_and *p*_1,*l *_are Gaussian density functions with the same variance. This means uncorrelated inputs are the same as independent inputs and that *p*_0,*l *_and *p*_1,*l *_are completely specified by three parameters: *μ*_0,*l*_, *μ*_1,*l*_, and σl2
 MathType@MTEF@5@5@+=feaafiart1ev1aaatCvAUfKttLearuWrP9MDH5MBPbIqV92AaeXatLxBI9gBaebbnrfifHhDYfgasaacH8akY=wiFfYdH8Gipec8Eeeu0xXdbba9frFj0=OqFfea0dXdd9vqai=hGuQ8kuc9pgc9s8qqaq=dirpe0xb9q8qiLsFr0=vr0=vr0dc8meaabaqaciaacaGaaeqabaqabeGadaaakeaaiiGacqWFdpWCdaqhaaWcbaGaemiBaWgabaGaeGOmaidaaaaa@30F6@. Under such assumptions, naïve Bayes becomes equivalent to Diagonal Linear Discriminant Analysis [[33], DLDA].

Other than using a penalized variance estimate for σl2
 MathType@MTEF@5@5@+=feaafiart1ev1aaatCvAUfKttLearuWrP9MDH5MBPbIqV92AaeXatLxBI9gBaebbnrfifHhDYfgasaacH8akY=wiFfYdH8Gipec8Eeeu0xXdbba9frFj0=OqFfea0dXdd9vqai=hGuQ8kuc9pgc9s8qqaq=dirpe0xb9q8qiLsFr0=vr0=vr0dc8meaabaqaciaacaGaaeqabaqabeGadaaakeaaiiGacqWFdpWCdaqhaaWcbaGaemiBaWgabaGaeGOmaidaaaaa@30F6@ (see below), we use the formulae derived by [33]. DLDA classifies an observation **x **to class 1 when

∑lvl≥0     (10)
 MathType@MTEF@5@5@+=feaafiart1ev1aaatCvAUfKttLearuWrP9MDH5MBPbIqV92AaeXatLxBI9gBaebbnrfifHhDYfgasaacH8akY=wiFfYdH8Gipec8Eeeu0xXdbba9frFj0=OqFfea0dXdd9vqai=hGuQ8kuc9pgc9s8qqaq=dirpe0xb9q8qiLsFr0=vr0=vr0dc8meaabaqaciaacaGaaeqabaqabeGadaaakeaadaaeqbqaaiabdAha2naaBaaaleaacqWGSbaBaeqaaOGaeyyzImRaeGimaadaleaacqWGSbaBaeqaniabggHiLdGccaWLjaGaaCzcamaabmaabaGaeGymaeJaeGimaadacaGLOaGaayzkaaaaaa@3AA5@

where

vl=μ1,l−μ0,lσl2(xl−μ0,l+μ1,l2).     (11)
 MathType@MTEF@5@5@+=feaafiart1ev1aaatCvAUfKttLearuWrP9MDH5MBPbIqV92AaeXatLxBI9gBaebbnrfifHhDYfgasaacH8akY=wiFfYdH8Gipec8Eeeu0xXdbba9frFj0=OqFfea0dXdd9vqai=hGuQ8kuc9pgc9s8qqaq=dirpe0xb9q8qiLsFr0=vr0=vr0dc8meaabaqaciaacaGaaeqabaqabeGadaaakeaacqWG2bGDdaWgaaWcbaGaemiBaWgabeaakiabg2da9maalaaabaacciGae8hVd02aaSbaaSqaaiabigdaXiabcYcaSiabdYgaSbqabaGccqGHsislcqWF8oqBdaWgaaWcbaGaeGimaaJaeiilaWIaemiBaWgabeaaaOqaaiab=n8aZnaaDaaaleaacqWGSbaBaeaacqaIYaGmaaaaaOWaaeWaaeaacqWG4baEdaWgaaWcbaGaemiBaWgabeaakiabgkHiTmaalaaabaGae8hVd02aaSbaaSqaaiabicdaWiabcYcaSiabdYgaSbqabaGccqGHRaWkcqWF8oqBdaWgaaWcbaGaeGymaeJaeiilaWIaemiBaWgabeaaaOqaaiabikdaYaaaaiaawIcacaGLPaaacqGGUaGlcaWLjaGaaCzcamaabmaabaGaeGymaeJaeGymaedacaGLOaGaayzkaaaaaa@5766@

The left-hand side of (10) is monotonically equivalent to the posterior probability (9).

To estimate σl2
 MathType@MTEF@5@5@+=feaafiart1ev1aaatCvAUfKttLearuWrP9MDH5MBPbIqV92AaeXatLxBI9gBaebbnrfifHhDYfgasaacH8akY=wiFfYdH8Gipec8Eeeu0xXdbba9frFj0=OqFfea0dXdd9vqai=hGuQ8kuc9pgc9s8qqaq=dirpe0xb9q8qiLsFr0=vr0=vr0dc8meaabaqaciaacaGaaeqabaqabeGadaaakeaaiiGacqWFdpWCdaqhaaWcbaGaemiBaWgabaGaeGOmaidaaaaa@30F6@, we use a penalized estimate, σ˜l2
 MathType@MTEF@5@5@+=feaafiart1ev1aaatCvAUfKttLearuWrP9MDH5MBPbIqV92AaeXatLxBI9gBaebbnrfifHhDYfgasaacH8akY=wiFfYdH8Gipec8Eeeu0xXdbba9frFj0=OqFfea0dXdd9vqai=hGuQ8kuc9pgc9s8qqaq=dirpe0xb9q8qiLsFr0=vr0=vr0dc8meaabaqaciaacaGaaeqabaqabeGadaaakeaaiiGacuWFdpWCgaacamaaDaaaleaacqWGSbaBaeaacqaIYaGmaaaaaa@3105@, which is similar in spirit to variance estimates used in [34]. In particular,

σ˜l2
 MathType@MTEF@5@5@+=feaafiart1ev1aaatCvAUfKttLearuWrP9MDH5MBPbIqV92AaeXatLxBI9gBaebbnrfifHhDYfgasaacH8akY=wiFfYdH8Gipec8Eeeu0xXdbba9frFj0=OqFfea0dXdd9vqai=hGuQ8kuc9pgc9s8qqaq=dirpe0xb9q8qiLsFr0=vr0=vr0dc8meaabaqaciaacaGaaeqabaqabeGadaaakeaaiiGacuWFdpWCgaacamaaDaaaleaacqWGSbaBaeaacqaIYaGmaaaaaa@3105@ = *s*_0 _+ σ˜l2
 MathType@MTEF@5@5@+=feaafiart1ev1aaatCvAUfKttLearuWrP9MDH5MBPbIqV92AaeXatLxBI9gBaebbnrfifHhDYfgasaacH8akY=wiFfYdH8Gipec8Eeeu0xXdbba9frFj0=OqFfea0dXdd9vqai=hGuQ8kuc9pgc9s8qqaq=dirpe0xb9q8qiLsFr0=vr0=vr0dc8meaabaqaciaacaGaaeqabaqabeGadaaakeaaiiGacuWFdpWCgaacamaaDaaaleaacqWGSbaBaeaacqaIYaGmaaaaaa@3105@     (12)

where *s*_0 _= 0.01 is a small positive constant and σ˜l2
 MathType@MTEF@5@5@+=feaafiart1ev1aaatCvAUfKttLearuWrP9MDH5MBPbIqV92AaeXatLxBI9gBaebbnrfifHhDYfgasaacH8akY=wiFfYdH8Gipec8Eeeu0xXdbba9frFj0=OqFfea0dXdd9vqai=hGuQ8kuc9pgc9s8qqaq=dirpe0xb9q8qiLsFr0=vr0=vr0dc8meaabaqaciaacaGaaeqabaqabeGadaaakeaaiiGacuWFdpWCgaacamaaDaaaleaacqWGSbaBaeaacqaIYaGmaaaaaa@3105@ is the standard pooled variance estimate. As noted by [34], the resulting estimate σ˜l2
 MathType@MTEF@5@5@+=feaafiart1ev1aaatCvAUfKttLearuWrP9MDH5MBPbIqV92AaeXatLxBI9gBaebbnrfifHhDYfgasaacH8akY=wiFfYdH8Gipec8Eeeu0xXdbba9frFj0=OqFfea0dXdd9vqai=hGuQ8kuc9pgc9s8qqaq=dirpe0xb9q8qiLsFr0=vr0=vr0dc8meaabaqaciaacaGaaeqabaqabeGadaaakeaaiiGacuWFdpWCgaacamaaDaaaleaacqWGSbaBaeaacqaIYaGmaaaaaa@3105@ is numerically more stable than σ˜l2
 MathType@MTEF@5@5@+=feaafiart1ev1aaatCvAUfKttLearuWrP9MDH5MBPbIqV92AaeXatLxBI9gBaebbnrfifHhDYfgasaacH8akY=wiFfYdH8Gipec8Eeeu0xXdbba9frFj0=OqFfea0dXdd9vqai=hGuQ8kuc9pgc9s8qqaq=dirpe0xb9q8qiLsFr0=vr0=vr0dc8meaabaqaciaacaGaaeqabaqabeGadaaakeaaiiGacuWFdpWCgaacamaaDaaaleaacqWGSbaBaeaacqaIYaGmaaaaaa@3105@.

### SVM parameter selection

Due to the heavy computational burden of fine-tuning SVM parameters for each of the 369 functional categories, we tuned the parameters to a subset of the categories. Namely, we applied the same parameter values to functional categories with similar levels of informativeness. To select the parameters for SVM, we relied on the more global performance measure, AUC. As stated in the "Performance Evaluation" section, AUC does not depend on the threshold *m *and it summarizes the entire range of trade-offs that can be achieved by a ranking algorithm.

We trained SVMs on ten functional categories chosen as follows: We sorted all 369 functional categories according to their informativeness or gene association levels (again, we refer to a functional category as more informative if fewer genes are associated with it). From that sorted list, we split the categories into ten groups based on the deciles. For example, the first group is comprised of the most informative functions and the tenth group is comprised of the least informative functions. To construct the "training set", we chose one function from each of these groups, and chose the SVM parameter values corresponding to the highest AUC value for each of them.

We tested all three kernels (linear, radial basis function, and polynomial) with diagfactor = {0.01, 0.02, 0.05, 0.1, 0.5, 1, 2, 5, 10}. For radial basis function (RBF) kernels, we tested widthfactor = {0.1, 0.2, 0.25, 0.5, 0.75, 1, 1.5, 2} and for polynomial kernels, we tested power = {2, 3} which corresponds to the degree of the polynomial kernel. For each of the ten training set categories, the AUC values were plotted in contour plots to determine the highest value. If this optimal parameter set was on the boundary of the tested parameter range, the range of the parameter values were expanded. This process was repeated until we found a range of parameters in which the optimal parameter set did not lie on the boundary of our contour plots. Additional values tested include diagfactor = {0.001, 0.005, 12, 15, 20} and widthfactor = {3, 5, 7}.

To test whether the fine-tuned parameters generalizes for other functional categories, we measured their performance on a separate "test set". From each of the ten groups described above, we selected five additional functional categories as the "test set". The AUC values of the test set areshown in Table 6. Two different sets of parameters were applied to the SVM:"default", which corresponds to Gist's default parameters (diagfactor = 0.1 and widthfactor = 1) with RBF kernels (we chose this kernel since it appear to perform well across all functions we tested); and "tuned", which corresponds to the parameter values fine-tuned to the training set.

Table 6 illustrates that there is little difference between using the default and the fine-tuned parameters with respect to AUC values. Using default parameters resulted in higher AUC values than using the tuned parameters in 5 out of 10 groups, and the mean AUC across all groups were the same. Since the Gist default parameters appear to be robust across different functional categories for this particular data set, we decided to use the default parameters to train the SVMs for all 369 categories in our experiments.

### FAM parameter selection

FAM formally has one parameter: a penalty on diagonal variances, *s*_0_, mentioned in section *The Naïve Bayes Method*. We have not performed an extensive optimization of this parameter and settled on *s*_0 _= 0.01, since it seems that FAM is not very sensitive to its value.

## Authors' contributions

RK was responsible for defining the problem and preparing the data. All authors contributed equally to formulating the general modeling paradigm, investigating different implementations, and writing the paper. For specific tasks, RK worked on the implementation of FAM; RS worked on SVM experiments; and MZ worked on performance evaluation.

## Supplementary Material

Additional File 1 **GO-BP annotations **Tab-delimited file with 3225 lines each with 370 columns, including gene names (first column) and GO accession numbers (first line), for 3224 genes and 369 GO-BP categories used in the text. Contains binary annotation entries. Click here for file

Additional File 2 **FAM predictions **Tab-delimited file with 3225 lines each with 370 columns, including gene names (first column) and GO accession numbers (first line), for 3224 genes and 369 GO-BP categories used in the text. Contains FAM prediction scores realized when a gene was part of the validation set. Click here for file

Additional File 3 **FAM predictions **Tab-delimited file with 3225 lines each with 370 columns, including gene names (first column) and GO accession numbers (first line), for 3224 genes and 369 GO-BP categories used in the text. Contains SVM prediction scores realized when a gene was part of the validation set. Click here for file

## Figures and Tables

**Table 1:** Illustration of performance metrics. A hypothetical table illustrating 4 possibilities in predicting *one *GO category.

	In top-ranked list	Not in top-ranked list	Total
Associated	a	b	s
Unassociated	c	d	p-s

Total	m	p-m	p

**Table 2:** cross-validated performance of FAM and SVM, averaged over all 369 GO functions. For each of the four C-V folds, the other three folds are used to train FAM and SVM. The performance metrics listed are *averaged *over all 369 GO functions.

**CV Fold**	**Sensitivity**_10%_		**Specificity**_10%_		**AUC**	
	**FAM**	**SVM**	**FAM**	**SVM**	**FAM**	**SVM**
1	0.503	0.501	0.905	0.905	0.784	0.783
2	0.482	0.480	0.905	0.905	0.769	0.773
3	0.518	0.509	0.905	0.905	0.797	0.791
4	0.485	0.478	0.905	0.905	0.769	0.764

**mean**	**0.497**	**0.492**	**0.905**	**0.905**	**0.780**	**0.778**

**Table 3:** pairwise comparison of predictions made by FAM and SVM for 369 GO functions in 4 cross-validation experiments. For each of the 369 GO functions, the performance of FAM and SVM are compared. "Win," "Tie" and "Lose" refer to the number of GO functions for which FAM's predictions are better than, tied with, or worse than that of SVM, respectively. The last row, denoted by "ACV," compares FAM and SVM using performance metrics which are averaged over the 4 cross-validation folds. As expected, this gives rise to fewer ties.

**CV Fold**	**Sensitivity**_10%_			**Specificity**_10%_			**AUC**		
	**Win**	**Tie**	**Lose**	**Win**	**Tie**	**Lose**	**Win**	**Tie**	**Lose**
1	106	168	95	107	167	95	193	2	174
2	87	181	101	88	183	98	177	1	191
3	104	171	94	109	166	94	186	3	180
4	90	193	86	92	192	85	199	6	164
ACV	184	36	146	194	34	141	201	0	168

**Table 4:** averaged cross-validation performance of FAM and SVM for 369 GO functions classified into 4 groups based on how informative they are. A GO function is said to be more informative if fewer numbers of genes are associated with it. N = number of GO functions in each group. H, MH, ML, L denote the 4 groups with high, medium-high, medium-low, and low levels of informativeness. For each GO function, the averaged cross-validation (CV) performance metrics of FAM and SVM are used. These (averaged) CV performance metrics are then averaged over all GO functions within each category. Notice that the last row is identical to that in Table 1.

		**Sensitivity**_10%_		**Specificity**_10%_		**AUC**	
**Group**	N	**FAM**	**SVM**	**FAM**	**SVM**	**FAM**	**SVM**
H	95	0.550	0.532	0.903	0.903	0.788	0.784
MH	144	0.521	0.521	0.904	0.904	0.792	0.790
ML	71	0.449	0.445	0.906	0.906	0.771	0.768
L	59	0.412	0.412	0.908	0.908	0.748	0.749

**mean**	369	**0.497**	**0.492**	**0.905**	**0.905**	**0.780**	**0.778**

**Table 5:** pairwise comparison of predictions made by FAM and SVM for 369 GO functions classified into 4 groups based on how informative they are. A GO function is said to be more informative if fewer numbers of genes are associated with it. N = number of GO functions in each group. H, MH, ML, L denote the 4 groups with high, medium high, medium low, and low levels of informativeness. For each GO function, the performance of FAM and SVM are compared using averaged performance metrics over the 4 cross-validation folds. "Win," "Tie" and "Lose" refer to the number of GO functions for which FAM's predictions are better than, tied with, or worse than that of SVM, respectively. Notice that the last row is identical to that in Table 2.

		**Sensitivity**_10%_			**Specificity**_10%_			**AUC**		
**Group**	N	**Win**	**Draw**	**Lose**	**Win**	**Draw**	**Lose**	**Win**	**Draw**	**Lose**
H	95	46	22	27	46	20	29	55	0	40
MH	144	64	12	68	76	12	56	74	0	70
ML	71	43	2	26	39	2	30	39	0	32
L	59	31	0	28	33	0	26	33	0	26

ACV	369	184	36	149	194	34	141	201	0	168

**Table 6:** AUC of SVM on the testing set. The testing set is comprised of 50 functional categories classified into 10 groups according to how informative they are. A GO function is said to be more informative if fewer numbers of genes are associated with it. An SVM was trained for each of these categories using two sets of parameters. In this table, Group 1 consists of the most informative functions whereas Group 10 consists of the least informative functions. The "default" column reports to the prediction performance on the AUC scale using default control parameters in our SVM software Gist. The "tuned" column reports the prediction performance on the AUC scale using control parameters optimized on the corresponding the training set. The AUC values listed are averaged across the four C-V folds. See the substraction "SVM" Parameter Selection" in the "Method" section for further detail.

Group	Default	Tuned
1	0.804	0.793
2	0.683	0.638
3	0.803	0.806
4	0.853	0.862
5	0.864	0.863
6	0.751	0.740
7	0.788	0.805
8	0.807	0.796
9	0.748	0.773
10	0.739	0.766

Mean	0.784	0.784
(Stdev)	(0.055)	(0.064)
